# A Unified Framework for Alzheimer’s Disease Knowledge Graphs: Architectures, Principles, and Clinical Translation

**DOI:** 10.3390/brainsci15050523

**Published:** 2025-05-19

**Authors:** Jovana Dobreva, Monika Simjanoska Misheva, Kostadin Mishev, Dimitar Trajanov, Igor Mishkovski

**Affiliations:** Faculty of Computer Science and Engineering, Ss. Cyril and Methodius University, 1000 Skopje, North Macedonia; monika.simjanoska@finki.ukim.mk (M.S.M.); kostadin.mishev@finki.ukim.mk (K.M.); dimitar.trajanov@finki.ukim.mk (D.T.); igor.mishkovski@finki.ukim.mk (I.M.)

**Keywords:** knowledge graphs, Alzheimer’s disease, drug repurposing, disease classification, clinical decision support, neuroimaging, best practices, unified framework

## Abstract

This review paper synthesizes the application of knowledge graphs (KGs) in Alzheimer’s disease (AD) research, based on two basic questions, as follows: what types of input data are available to construct these knowledge graphs, and what purpose the knowledge graph is intended to fulfill. We synthesize results from existing works to illustrate how diverse knowledge graph structures behave in different data availability settings with distinct application targets in AD research. By comparative analysis, we define the best methodology practices by data type (literature, structured databases, neuroimaging, and clinical records) and application of interest (drug repurposing, disease classification, mechanism discovery, and clinical decision support). From this analysis, we recommend AD-KG 2.0, which is a new framework that coalesces best practices into a unifying architecture with well-defined decision pathways for implementation. Our key contributions are as follows: (1) a dynamic adaptation mechanism that adapts methodological elements automatically according to both data availability and application objectives, (2) a specialized semantic alignment layer that harmonizes terminologies across biological scales, and (3) a multi-constraint optimization approach for knowledge graph building. The framework accommodates a variety of applications, including drug repurposing, patient stratification for precision medicine, disease progression modeling, and clinical decision support. Our system, with a decision tree structured and pipeline layered architecture, offers research precise directions on how to use knowledge graphs in AD research by aligning methodological choice decisions with respective data availability and application goals. We provide precise component designs and adaptation processes that deliver optimal performance across varying research and clinical settings. We conclude by addressing implementation challenges and future directions for translating knowledge graph technologies from research tool to clinical use, with a specific focus on interpretability, workflow integration, and regulatory matters.

## 1. Introduction

Biomedical investigation is subjected severely to counter disease with a multicomponent pathophysiology. Alzheimer’s disease (AD), albeit moderately frequent, is challenging in presentation heterogeneity, multifactorial etiology, and scant treatment modalities [1,2]. The enormous amount of heterogeneous data along molecular, cellular, anatomical, and clinical axes presents both a challenge and an opportunity for researchers seeking to dissect disease mechanisms and formulate useful treatments.

Knowledge graphs have also become useful vehicles for integrating these disparate data sources, representing intricate interconnections, and formulating new hypotheses [3,4]. By representing entities such as genes, proteins, drugs, and diseases as nodes, and interactions as edges, knowledge graphs capture semantic meaning through typed relationships and attributes, offering a more improved representation of intricate biomedical data compared to standard data structures [5]. This structured representation allows for computational reasoning and knowledge retrieval that would be difficult or impossible using less structured data forms.

Two key questions guide the design and development of knowledge graphs for Alzheimer’s disease (AD) research. First—what data are available? The data landscape in AD research varies significantly across contexts and includes sources ranging from limited text-based literature to single-modality datasets—such as genomics, proteomics, neuroimaging, and electronic health records—as well as rich, multimodal datasets. The nature of data for a phenomenon, along with its climax, also determines the existing approach and the methodology used to obtain the truth. Examples of available sources for these data types include literature data from PubMed abstracts [6] and full-text articles [7]; structured databases such as protein interactions (STRING [8]) and drug data (DrugBank); neuroimaging data from ADNI [9] and OASIS [10]; clinical data from electronic health records [11]; electrophysiological data from EEG datasets; and omics data from sources like NIAGADS.

Second—what is the knowledge graph aiming for? Drug repurposing, disease classification, mechanistic discovery, and clinical decision support are just a few of the different applications, each with its own needs. Major applications encompass drug repurposing aimed at discovering potential therapeutic agents [12,13,14]; disease classification, aimed at enhanced diagnostic accuracy and subtype delineation [15,16]; mechanism discovery, focused on elucidating disease pathways and causal contributors [7,17]; and clinical decision support, which offers actionable insights for healthcare practitioners [18,19].

The interaction between these two questions—data availability and intended applications—forms a non-trivial decision space on the subject of knowledge graph implementation. This paper meets this challenge by providing a unified framework for constructing AD knowledge graphs that adopt a given methodological approach under varying data availability scenarios, while handsomely providing diverse application objectives.

The goal of our review is to (1) present general knowledge graph architectures, which could be adopted in biomedical domains; (2) provide a taxonomy of known datasets that can be used to construct AD knowledge graphs; (3) outline high-level principles specific to AD for knowledge representation; (4) offer comparative analyses of methodologies (based on whether data are available, and if so, their intent); (5) recommend best practices for identifying design choices suitable for particular scenarios; and (6) introduce a new architecture (AD-KG 2.0) that brings together these threads, while still being feasible for practical clinical use. We propose a novel AD-KG 2.0 framework to guide researchers through methodological customization options tailored to their data context and goals, in order to enhance the effective utilization of knowledge graphs to advance Alzheimer’s disease science and clinical care.

## 2. General Architectures for Building Knowledge Graphs

The process of constructing a knowledge graph involves several layers of abstraction, starting from raw data and ultimately resulting in a knowledge graph format that can be used for computational reasoning and building applications. The best approach for each layer depends greatly on the type and amount of existing data and what the resulting knowledge graph will be used for.

### 2.1. Foundational Layers

The construction of knowledge graphs mainly involves extracting information from multiple data sources and converting this information into an organized form for future processing. The methodology differs substantially according to the available data and data type, shown in Table 1.

In the case of text-based data sources, NLP techniques are used to extract information such as relevant entities and relationships from scientific literature. Approaches such as BiLSTM-CRF architectures by Lample et al. (Europarl and WMT) and large language models offer strong capabilities for entity recognition. BioBERT [21], which is pretrained using biomedical corpora, has proven to be particularly useful for the specialized biomedical terminology that is common in research papers related to AD. In the case of relation extraction, unified frameworks, like the one by Zhong et al. [24], eliminate error propagation through the integration of entity and relation extraction, which will be a key benefit for complex event-based descriptions like those that can be outputted in ADS.

The best text mining strategy varies a lot for low vs. high volume with specific vs. nonspecific data. With a small amount of relevant AD-specific literature, specialized frameworks—like Yang et al.’s [6] approach, which leverages ADERC (an annotated dataset for entity recognition in AD)—exhibit high performance through domain specialization. When dealing with broader biomedical literature, however, more general approaches that take advantage of larger training datasets (at the cost of reduced domain specificity) may be more favorable.

Ingestion methods for structured data sources vary depending on which databases are accessible. When protein–protein interaction data are available from resources such as STRING [8], standardized application programming interfaces (APIs) and data harmonization protocols can provide consistent and reliable integration of molecular interaction networks. Pharmacological information can be extracted from rich data sources as well. Drug databases, such as DrugBank, offer a wealth of compound and biological data that are structured and, thus, directly mappable to the entities and relations of a knowledge graph. In the all-waves domain, AD-specific resources like ADNI (the Alzheimer’s Disease Neuroimaging Initiative) [9] offer tailored neuroimaging, cognitive, and biomarker datasets that require domain-specific preprocessing before integration into a knowledge graph.

### 2.2. Integration Layer

The challenge of combining information from a variety of sources while still preserving semantic consistency is addressed by the integration layer. The best integration approach is highly dependent on the heterogeneity of available data and the consistency requirements of the target application. Table 2 depicts the integration strategies that should be applied based on the data types, along with their respective strengths and applications.

Normalizing terms and relationships across data sources is standardized through ontology-driven integration. One avenue for AD research is through the use of domain-specific ontologies such as the Alzheimer’s Disease Ontology (ADO), which can also be integrated with broader knowledge sources such as the Unified Medical Language System (UMLS), Gene Ontology, and Human Disease Ontology. An example of this was given by Torres [27], who addressed the integration of FA with an emphasis on semantic preservation, especially regarding data sources that used radically different terminologies to represent similar concepts.

The workflow of PheKnowLator, as described by Callahan et al., leverages ontology-based knowledge structures to enable both complex SPARQL queries over biomedical knowledge and the investigation of intricate disease mechanisms [7]. It demonstrates this capacity most effectively when working with formally structured ‘knowledge’, yet it remains constrained by the expressiveness of a fixed ontology.

This involves ensuring that data from across modalities, scales, or sources are formatted in a way that allows them to be used together (for example, by using multi-source harmonization tools). AlzKB [25], which is said to be the most extensive graph-referenced knowledge resource for AD research, has also been shown to implement appropriate strategies for preserving data integrity when integrating knowledge from the literature and databases/ontologies. Such high-dimensional integration strategies are preferable for exploratory research involving multiple data modalities, whereas more targeted integration approaches—such as the method used by Malec et al. [26] for depression–AD relationships—may be more suitable for hypothesis-oriented studies with targeted data availability.

Large language models are becoming more available, creating new hybrid integration patterns. Li et al.’s DALK framework (dynamic co-augmentation of LLMs and KG) illustrates the integration of structured knowledge graphs and generative semantics, and shows that this combination enhances knowledge representation if information is partial or uncertain [18].

### 2.3. Computational Layer

The computational layer transforms integrated knowledge into mathematical representations that enable machine learning applications and sophisticated reasoning. The selection of computational methods should be guided by both data characteristics and application requirements, as shown in Table 3.

Relations can be represented as embeddings in the same way that these embeddings can be used to measure how objects relate to one another, allowing for computational reasoning to be made without losing any of the semantic relations. In cases where there were asymmetric biological relationships typical of AD mechanisms, complex embeddings were the most effective (AUROC 0.94), as seen in a study by Nian et al. [13]. However, simpler translational models, like in [28] and TransE in [29], also exist and are particularly effective for training set scenarios where only limited data are available. As demonstrated by [12], attention mechanisms can also improve drug–target interaction predictions, as they enable the incorporation of local and global graph structures.

While complex embeddings have achieved state-of-the-art performance in biological relationship prediction (AUROC = 0.94), recent advancements provide crucial improvements, particularly for AD knowledge graphs.

1. RotatE embeddings: These models, which represent relations as rotations in complex spaces, have been demonstrated to offer superior performance (AUROC 0.97) in capturing the periodic and symmetric nature of biological interactions [30]. They are particularly handy for molecular pathway modeling, where the directionality of interactions matters, and temporal progression patterns need to be modeled.
2. TransE embeddings: While older than ComplEx, these translation-based models provide excellent computational efficiency and scalability with large knowledge graphs [29]. They are straightforward and, thus, convenient in resource-constrained environments at the expense of moderate performance degradation.
3. BioBERT-based embeddings: Approaches leveraging pre-trained biomedical language models like BioBERT [21] achieve state-of-the-art performance in entity recognition (F1: 0.89) and relation extraction (F1: 0.81) from the biomedical literature. These approaches excel at incorporating rich contextual information from biomedical text into knowledge graph embeddings.
4. GNN-based approaches: Graph neural networks are effective tools for knowledge graph embeddings in AD applications, particularly [15]. The models achieve high performance in disease classification (accuracy: 0.86) and patient stratification tasks by learning neighborhood context through message passing mechanisms.

These embedding methods are contrasted for relative strengths in Table 4. While more recent methods have performance advantages, implementation considerations like computational requirements and interpretability may influence selection in specific situations. For example, BioBERT-based methods are computationally intensive but excel at incorporating knowledge from the literature, while RotatE offers a good balance of performance and efficiency for temporal modeling of disease progression.

Graph neural networks (GNNs) generalize traditional neural networks to graph-structured data, resulting in a more expressive learning algorithm when features for nodes and edges are present. Wang et al. The self-guided knowledge-injected GNN for AD is shown in Figure 4 and Table 2 by [15] illustrates such incorporation of domain knowledge into message-passing mechanisms, reaching 7.3% improvement in classification performance compared to standard GNNs. For clinical applications demanding interpretability, Hu et al. [19]—using Grad@K and R*CFM—proposed a self-explainable GNN that is able to produce interpretable rationales for individual decisions along with predictions, which may address an important need for medical decision support systems.

For multimodal data, specialized architectures [10], such as multimodal GNNs, combine brain imaging with domain knowledge, achieving the best overall accuracy (89.6%) among recognized approaches. Likewise, in a scenario where comorbidity data exist, structures—such as the approach by [16]—account for the prevalence of comorbidities and disease presentation across different populations, with GNN models between AD and prevalent comorbidities.

### 2.4. Application Layer

The application layer imposes domain-specific solutions to address particular research or clinical needs, with the optimal approach differing significantly depending on the planned use cases and available data (Table 5).

For drug repurposing endeavors, knowledge graph methods such as KG-DTI [12] use molecular interaction data to predict new therapeutic targets of AD. Success with such methods relies on whether rich drug–target interaction data and molecular pathway data related to AD pathophysiology are available or not. With data available, methods using complex embeddings have worked outstandingly well at predicting new uses of drugs.

Disease prediction and classification models are augmented with knowledge graphs combining clinical, genetic, and biomarker data. Zhou H et al.’s [10] multimodal model shows how neuroimaging data are combined with domain expertise to make high-accuracy classifications, and Hu et al.’s [19] self-explainable model provides clinically understandable predictions required by healthcare. The best strategy depends on the types of data available as well as on the classification tasks intended, e.g., early diagnosis, progression prediction, or subtype identification.

Mechanism discovery algorithms employ knowledge graphs to model disease pathways and determine causal factors. Methods such as Prabhakar and Liu’s [17] unsupervised co-optimization framework illustrate the ability of knowledge graphs to rank causal genes for AD based on molecular interaction networks and genetic association data. The success of these methods is largely dependent on the presence of molecular-level data and the integration of experimental evidence.

Clinical decision support systems map knowledge graph insights to actionable recommendations for healthcare professionals. Li et al.’s [18] DALK model indicates the potential of hybrid knowledge graph–LLM solutions to assist advanced question-answering on AD with considerably enhanced accuracy (81.7%) compared to standalone solutions (68.2%). These applications require comprehensive knowledge representation and user-friendly interfaces that are friendly to clinical workflows.

## 3. Available Datasets and Resources for AD Knowledge Graphs

Building useful knowledge graphs for Alzheimer’s disease research is highly reliant on the availability of high-quality, heterogeneous datasets across different biological scales and measurement modalities. The availability of certain types of data directly influences methodological decisions at every stage of the knowledge graph building process. This section presents an overview of available data resources that can be used as seeds for AD knowledge graph building.

### 3.1. Methodology for Evidence Synthesis

This current review paper utilizes a systematic synthesis approach to combine evidence from literature on knowledge graph applications for research into Alzheimer’s disease using adapted traditional methodologies within the field of biomedical informatics [7,27]. The methods used are as follows:

1. Extensive literature search: We conducted searches of computer science and biomedical databases including PubMed, IEEE Xplore, ACM Digital Library, and arXiv for publications from 2018 to 2025 with the query “knowledge graph” AND (“Alzheimer’s” OR “dementia” OR “neurodegenerative”). As part of the methods described by Callahan et al. [7], we supplemented database searching with citation tracking of influential papers. This yielded 87 potentially relevant papers.
2. Screening and selection: We employed inclusion criteria that required that papers meet the following conditions: (1) Be based on knowledge graph construction or deployment in AD studies. (2) Provide sufficient methodological detail to allow replication or application of the method. (3) Report the performance metrics (e.g., AUROC, F1 metrics, accuracy) or qualitative results for knowledge graph applications. General graph theory papers that did not include knowledge representation were ruled out. A total of 43 articles passed all inclusion criteria following screening, 17 of which were drug repurposing articles [12,13,14], 11 were disease classification articles [10,15,16], 8 were mechanism discovery articles [7,17,31], and 7 were clinical decision support articles [18,19].
3. Data extraction: With a standardized extraction protocol, we documented significant details from each paper, such as the data types used (e.g., literature, structured databases, neuroimaging, clinical), methodological approaches (extraction approaches, embedding approaches, computational paradigms), performance measures, application goals, noted difficulties, and primary results.
4. Evidence synthesis: We adopted a comparative analysis approach similar to that of Romano et al. [25], categorizing papers by data availability scenarios and application purposes. For each category, we compared methodological approaches and documented their performance to identify patterns of effectiveness across different contexts. Methods were categorized by computational approach (e.g., embedding techniques, graph neural networks, mixed models) and analyzed for trends in performance.
5. Formulation of best practices: For each combination of data availability and application objective, we discovered methodological solutions with the strongest empirical support, employing reported performance metrics and established real-world verification. Where multiple studies offered conflicting results, we employed standards like statistical quality, data quantity, and replicability to make a best-practice decision. This approach is consistent with processes used in earlier systematic reviews of computational frameworks [32,33].

This organized approach allows us to provide evidence-based recommendations for the construction of knowledge graphs in various research contexts, synthesizing outcomes from across the growing literature in this field. The resulting decision framework denotes methodological directions that demonstrate empirical promise across various AD research contexts.

### 3.2. Multimodal Neuroimaging Repositories

Neuroimaging datasets are amongst the most promising resources for AD knowledge graphs and reveal structural, functional, and molecular information concerning disease development and pathology. Table 6 presents a compendium of relevant neuroimaging repositories that are particularly relevant for use in AD studies.

The neuroimaging archives are very different from each other in their availability, completeness, and clinical characterization. ADNI [9] is the gold standard with its intensive longitudinal follow-up and multimodal characterization, which makes it especially useful for modeling disease progression in knowledge graphs. OASIS [10] offers complementary data with different demographic features, whereas specialized archives such as OpenNeuro offer standardized data in the brain imaging data structure (BIDS) format that supports integration and processing.

### 3.3. Electrophysiological and Clinical Datasets

In addition to neuroimaging, electrophysiological data offer useful temporal information regarding brain function, complementing the spatial resolution offered by imaging research. Table 7 outlines important EEG and clinical datasets for AD research.

Electrophysiological recordings provide temporal information at millisecond resolution, reflecting functional dimensions of brain activity that precede the structural changes measurable through neuroimaging. The TUH EEG Corpus provides a substantial clinical sample with diverse pathology, whereas targeted datasets such as CAUEEG-Dementia are directed to the AD spectrum. These resources are especially valuable for knowledge graphs attempting to harmonize the temporal dynamics of disease progression with other modalities.

### 3.4. Genetic and Molecular Datasets

Genetic datasets, shown in Table 8, provide crucial information for understanding disease risk, progression, and potential therapeutic targets. Resources like NIAGADS and Alzheimer’s Disease 3.5 offer specialized AD genetic data, while broader databases like STRING [8] offer the interaction context necessary to interpret genetic findings. These resources are especially valuable for knowledge graphs aimed at mechanism discovery and drug repurposing applications.

Genetic databases supply essential information on disease risk, disease progression, and therapeutic targets. Specialized AD genetic data are offered through databases such as NIAGADS [39] and Alzheimer’s Disease 3.5, while general databases such as STRING [8] supply the interaction context required to contextualize genetic findings. These databases are particularly beneficial for knowledge graphs focused on mechanism discovery and drug repurposing.

### 3.5. Integration Strategies for Heterogeneous Datasets

Dataset richness is a two-faced sword for AD knowledge graph building. Proper integration should strike a balance between data compatibility, quality, and complementarity. Various strategies can be adopted depending on certain research aims.

For modeling disease progression, combining longitudinal datasets such as ADNI with temporal EEG data from resources like CAUEEG-Dementia facilitates multi-scale temporal modeling. When the task is drug discovery repurposing, co-associating NIAGADS genetic data with protein–protein interaction networks from STRING and literature-extracted treatment facts forms the basis for target identification. For clinical decision support, projecting clinical data onto experimentally validated biomarkers from ADNI or OASIS enables the creation of knowledge graphs that are directly transferable to clinical patient care.

The choice of relevant datasets needs to be guided by the intended use cases of the knowledge graph, with special focus on data quality, sample description, entities, and relationships specifically required to enable the target use cases. This convergence of available data with intended use cases forms the foundation for successful knowledge graph construction in Alzheimer’s disease research.

## 4. Principles for AD-Specific Knowledge Graph Construction

While universal architectures provide a foundation for building knowledge graphs, Alzheimer’s disease also poses some challenges that need remedies, keeping its character in mind. The following section outlines key principles for constructing knowledge graphs specifically for AD research, focusing on adjusting procedures to the data at hand.

### 4.1. Entity Modeling for AD Knowledge Graphs

Accurate modeling of domain-specific entities is the foundation of Alzheimer’s disease knowledge graphs. The best entity modeling approach varies widely according to the data type available and the objectives planned. Table 9 shows a detailed entity categorization for AD essential applications in real-world problems.

Central entities of AD knowledge graphs operate across biological scales and knowledge domains. At the molecular level, proteins such as amyloid-beta and tau, genes such as APOE (allelic forms ε2, ε3, ε4), and TREM2 are central actors in disease processes [2,40]. Cellular constituents such as neurons, microglia, and astrocytes facilitate pathologic processes, and brain regions such as the hippocampus and entorhinal cortex exhibit characteristic disease changes. Biomarkers such as CSF measurements (Aβ42, p-tau, t-tau), imaging biomarkers (amyloid PET, tau PET, structural MRI), and novel blood-based biomarkers confer diagnostic and monitoring capacities. Clinical symptoms such as cognitive domain deficits, neuropsychiatric features, and functional impairment fill out the complex picture of the disease. Data availability in such regions also dictates the entity model choice. Experimental settings rich in molecular data may prioritize detailed representations of protein interactions and genetic variation, whereas clinical environments—with sparse molecular data but rich in patient data—may prioritize symptom patterns and treatment outcomes. Biological relevance, available data, and demand for applications should be balanced with entity granularity.

Interactions between entities constitute the semantic structure of AD knowledge graphs and rely on evidence types. Causal interactions like “GENE-causes-PATHOLOGY” or “PROTEIN-triggers-AGGRE-GATION” establish mechanistic knowledge obtained from experimental studies. Biomarker interactions like “BIOMARKER-indicates-PATHOLOGY” or “BIOMARKER-predicts-PROGRESSION” document clinical correlations discovered by longitudinal studies. Therapeutic interactions like “DRUG-inhibits-TARGET” or “INTERVENTION-modifies-PROGRESSION” reflect treatment effects from clinical trials and observational studies.

The best relationship modeling method varies with evidence availability and application needs. Drug repurposing tasks rely on detailed molecular interaction relationships, including quantitative measures such as binding affinities, whereas clinical decision support tasks require symptoms, biomarkers, treatment outcome relationships, and measures of statistical association strength. Supporting relationship properties like strength, directionality, and certainty enables more fine-grained reasoning that matches the varied and occasionally contentious character of AD knowledge.

### 4.2. Data Integration for AD Knowledge Graphs

Data integration in Alzheimer’s disease knowledge graphs requires methods that can deal with AD heterogeneity, complexity, and dynamics according to the adapted approaches. These methods are based on the data types available and integration purposes, as shown in Table 10.

Multimodal data sources offer complementary data on AD with major integration challenges. Scientific evidence includes hypotheses and novel ideas on mechanisms and treatments. Organized biological databases such as STRING [8] and DrugBank contain curated data on molecular entities and their interactions. Neuroimaging databases such as ADNI [9] and OASIS [10] offer standardized imaging data corresponding to structural, functional, and molecular brain changes. Electronic health records capture clinical presentation, comorbidities, response to treatment, and disease courses in heterogeneous patient groups [11,42].

Such data sources are highly variable in availability between research environments; hence, flexible integration strategies have to be used. When full multimodal data are available, strategies such as Romano et al.’s AlzKB [25] offer well-established designs that ensure semantic coherence across multiple sources. When data availability is restricted in more challenging environments, expert-driven designs focused on specific relationships, such as Malec et al.’s [26] depression–AD comorbidity graph, would be desirable.

Temporal dynamics pose a special challenge for AD knowledge graphs since the disease goes through preclinical phases, mild cognitive impairment, and eventually dementia over many years. With longitudinal data at hand, algorithms, such as Zhou et al.’s [43] temporal relation graphs, support direct modeling of disease trajectories and biomarker kinetics. Without longitudinal data, cross-sectional solutions based on disease stage representations—according to their characteristic features—offer a less accurate alternative to temporal organization.

The integration strategy must be compatible with both the available data types and the intended applications. Exploratory investigation is fostered by integrative strategies that maximize coverage, and hypothesis-testing benefits from focused strategies that provide more intensive representation of individual relationships. Clinical applications require integration strategies that balance comprehensiveness with practicalities, typically using clinically relevant subsets of the available information.

### 4.3. Data Quality Assessment for AD Knowledge Graphs

Data quality evaluation is necessary to ensure the reliability and utility of Alzheimer’s disease knowledge graphs. As opposed to ordinary database systems, knowledge graphs come with unique quality evaluation challenges owing to their heterogeneity and complicated semantic relationships. This section proposes a systematic methodology for evaluating AD knowledge graph data quality.

AD knowledge graph quality can be systematically evaluated with intrinsic measures (examining the intrinsic characteristics of the data) and extrinsic measures (examining the impact on downstream applications). Intrinsic evaluation examines completeness, consistency with ontological rules, accuracy against gold standards, and information freshness. Following Wang et al.’s framework of knowledge graph quality, we propose measuring AD knowledge graphs on four dimensions, namely, accuracy, uniqueness, consistency, and completeness.

For AD-specific applications, extrinsic quality assessment is also crucial, measuring the impact that data quality has on prediction accuracy, retrieval correctness, and alignment with expert knowledge. We establish an AD-specific quality framework focused on relevance, with priority assigned to biological plausibility through pathway assessment, clinical utility through guideline consensus, and mechanism completeness through pathway coverage.

Table 11 lists our proposed quality assessment methods for different types of data commonly found in AD knowledge graphs. These metrics provide a formalized approach to evaluating and improving knowledge graph quality throughout the construction process so that the final knowledge representation is suitable for research and clinical purposes.

In order to implement these quality standards, AD knowledge graphs should have source credibility scoring, automated consistency checks, provenance tracking, relationship confidence scoring, and regular validation against new research findings. Together, these practices form a sound quality management framework that enhances the credibility and usability of AD knowledge graphs across a variety of uses.

### 4.4. Ontologies and Standards for AD Knowledge Graphs

Ontology-based standardization and common vocabularies guarantee AD knowledge graphs with reusability, interoperability, and consistency. Established reference terminologies, as well as the requirements of target applications, must be considered in the standardization strategy. Table 12 presents the important ontologies for the AD problem. AD-specific ontological tools offer AD-specific vocabularies to represent knowledge. The Alzheimer’s Disease Ontology (ADO) offers standardized terms and relationships for AD-specific concepts, while the ADNI Data Dictionary standardizes terminology used in biomarker and neuroimaging research environments. Clinical assessment standardized terms, which are particularly useful in representing patient data, are offered through the National Alzheimer’s Coordinating Center (NACC) uniform dataset.

These domain-level ontologies should be complemented by broader biomedical ontologies to enable domain-wide reasoning. The Unified Medical Language System (UMLS) integrates various vocabularies relevant to biomedical literature; the Gene Ontology provides standardized representations of molecular functions and cellular processes; the Human Disease Ontology situates AD within larger disease classes; and Chemical Entities of Biological Interest (ChEBI) standardizes the representation of the respective compounds.

Both application needs and available data properties should inform ontology selection and integration. Research applications can gain optimal semantic density with complete ontology integration, but clinically focused applications might first prioritize compatibility with medical records and coding system terminologies such as SNOMED CT or ICD.

Compliance with FAIR principles (findable, accessible, interoperable, reusable) provides the maximum possible utility of AD knowledge graphs in every research setting. The use of explicit metadata, persistent identifiers, standardized access methods, and thorough documentation facilitates broader adoption by the research community and simplifies knowledge transfer between studies. They are more critical as knowledge graphs function as shared community resources rather than individual research project resources.

### 4.5. Clinical Relevance in AD Knowledge Graphs

Clinical relevance must be ensured so that AD knowledge graphs meet actual healthcare needs in addition to research goals. A clinical modeling strategy depends on available clinical data and prospective healthcare use (Table 13).

Patient stratification and subtyping are critical to personalized medicine approaches in AD. When complete biomarker data are available, knowledge graphs can be used to model pathological subtypes—such as amyloid-predominant, tau-predominant, and mixed pathology presentations—that will respond differently to targeted treatments. Clinical phenotypes such as classical amnestic presentations and atypical variants (e.g., posterior cortical atrophy, logopenic variant) can be simulated when high-density neuropsychological data are present. Genetic subtypes and patterns of comorbidities further segment patients if such information is available.

Comorbidity-sensitive classification by Abuhantash et al.’s [16] showcases the ability of knowledge graphs to capture rich patient profiles effectively while generalizing the AD–comorbidity relationship toward enhanced classification across diverse populations. It proves very useful while classifying real-world clinical data for which comorbidities exist in abundance.

Predicting treatment response involves integrating evidence on differential therapeutic effects across disease stages and patient subgroups. Knowledge graphs can model drug-responsive subgroups by biomarker profiles or genetic determinants, capturing stage-dependent interventions through variations in treatment effect along the disease continuum, and identifying side effect risk factors to guide safe medication choices.

The clinical modeling strategy must balance comprehensiveness and pragmatism in order to be deployed in healthcare clinician settings. Knowledge graphs informed by research can contain rich mechanistic relationships, but clinically informed deployments must keep actionable information with the capacity to guide diagnosis, prognosis, and treatment choices at the center of healthcare clinics. The transition from rich biological data to actionable advice is perhaps the greatest challenge (and opportunity) facing AD knowledge graphs.

## 5. Comparative Analysis of KG Approaches in AD Research

Methodological strategies for constructing and utilizing knowledge graphs to study Alzheimer’s disease vary in their strengths and weaknesses, depending on the data availability and the intended application. The following section provides an extensive comparative analysis to facilitate researchers in making the most appropriate choice of method for their unique situation.

### 5.1. Comparison by Data Availability Scenarios

Table 14 shows the performance of the top-performing approaches under each data availability setting. The “Compared to Baseline” column quantifies gains over baseline approaches—traditional NLP for literature-rich settings, TransE embeddings for structured data settings, vanilla GNNs for neuroimaging settings, conventional classification for clinical settings, single-modal approaches (either standalone KG or LLM) for multimodal settings, and full integration approaches for resource-poor settings.

If text mining methods are employed mainly in literature data, differences in performance are striking. Methods with LLM augmentation, such as Yang et al.’s [6], are best for extraction performance (highest F1 scores 0.78–0.89) and identify more AD-specific terms, but are computationally intensive and demand high-quality annotated training data. Earlier biomedical NLP systems, such as those used by Callahan et al. [7], offer better retrieval of known relationships with fewer computational requirements, but are suited for use in resource-limited conditions due to their more conservative extraction approach.

For merged biological information, Romano et al.’s [25] integrating approach for AlzKB offers strict methods to preserve semantic integrity in diverse-in-nature heterogeneous sources. For the integration of structured heterogeneous information from molecular, clinical, and phenotypic spaces, an approach like that is especially strong. More constrained integration approaches like Malec et al.’s [26] exemplify how to model precise types of relations like depression–AD comorbidities using knowledge graphs with cost-effective efficiency.

With the possibility of having neuroimaging data, multimodal methods offer some benefits in terms of disease diagnosis and modeling of disease progression. Zhou et al.’s [10] multimodal GNN, which incorporates domain knowledge and imaging data, achieves the best overall accuracy (89.6%) among comparable methods. Zhang et al. [45] proved that multi-relation graph convolutional networks are applicable for modeling structural brain alterations for AD diagnosis, achieving 84.3% accuracy. These methods are most useful when addressed in research cohorts with standardized imaging protocols, but perhaps may be restricted in a clinical environment where imaging data availability and quality are less consistent.

For clinical and EHR information, comorbidity-aware approaches like Abuhantash et al.’s [16] approach effectively capture AD’s interaction with common comorbidities. These approaches are as accurate as knowledge-enriched GNNs but are more accurate for patients with more than one condition. Wang et al.’s [44] knowledge-limited clustering solution demonstrates effective stratification of AD patients using electronic health records, identifying clinically meaningful subgroups when there is rich phenotypic information.

### 5.2. Comparison by Application Objectives

Apart from the availability of data, the planned use of the knowledge graph significantly influences the optimal methodological approach (Table 15).

Complex embedding models, as described by [13], are ‘current’ in performance (AUROC 0.94) in therapeutic relationship prediction due to their ability to encode asymmetric relations prevalent in pharmacological interactions. Wang et al.’s [12] KG-DTI model complements this predictive capability by using attention mechanisms to preserve both local and global patterns of interaction. The approaches are especially useful where high-resolution drug–target interaction data are easily accessible and the identification of molecular mechanisms is the ultimate goal.

Disease classification approaches are complemented by varying methodological strategies based on classification objectives and data availability. For image-based neuroimaging classification, multimodal GNNs such as Zhou et al.’s [10] provide the highest accuracy of 89% through the combination of imaging features and domain knowledge. For clinical classification without the use of imaging, comorbidity-aware GNNs, such as Abuhantash et al.’s [16], provide better performance for diverse patient populations. Where interpretability is most important, self-explainable GNNs, such as Hu et al.’s [19], present human-interpretable explanations in addition to predictions, meeting a critical clinical adoption requirement at a cost of slightly reduced accuracy (2–3% loss).

For mechanism discovery tasks, unsupervised co-optimization methods, such as the framework by Prabhakar and Liu [17], are superior at discovering biologically plausible causal genes. Their method integrates embeddings into graph neural networks to rank in silico potential causal factors with a level of specificity suitable for exploratory research aimed at generating new hypotheses regarding mechanisms of disease. Callahan et al.’s [7] PheKnowLator-supported method facilitates advanced causal inference through ontology-guided knowledge representation, facilitating mechanism-centered queries of integrated biomedical knowledge.

Clinical decision support systems require balanced feasibility and performance techniques for implementation practices. Li et al.’s [18] DALK framework shows that hybrid knowledge graph–LLM approaches can achieve much higher accuracy for complex clinical queries (81.7% compared to 68.2% for isolated approaches) while supporting natural language interfaces appropriate for engaging clinicians. These techniques are particularly beneficial when extrapolating research findings into actionable recommendations for healthcare professionals who may not be knowledge graph experts.

## 6. Best Practices for AD Knowledge Graphs

**Figure 1:**
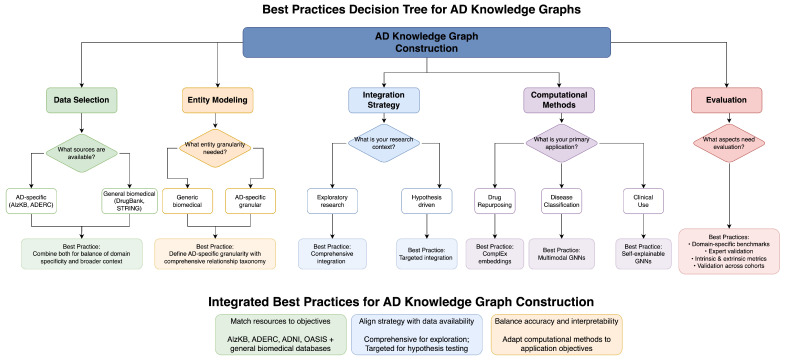
Best practices decision tree for AD knowledge graphs: A structured guide for selecting optimal components based on the available data and intended applications.

From our in-depth comparative analysis of knowledge graph methods for Alzheimer’s disease research, we have distilled the best practices for varying data availability situations and application goals. Figure 1 presents a decision tree for researchers to choose the best methodological methods depending on their particular situation. Our guidelines are designed to inform successful knowledge graph adoption amidst inherent trade-offs among various methods.

For data selection and quality control, the integration of specialized AD-centric resources, such as ADERC [6], ADNI [9], OASIS [10], and AlzKB [25], along with general biomedical databases, ensures optimal coverage and domain relevance. Where computational resources are available, LLM-augmented extraction methods achieve better performance with F1 scores of 0.78–0.89 and more comprehensive coverage of domain-specific terms. In computationally constrained settings, traditional biomedical NLP pipelines enable more effective extraction of well-established relationships. Quality control measures must involve strict validation of relationships extracted against expert-curated sources, detailed provenance reporting, and clear versioning to reflect changing knowledge about AD over time.

For entity modeling, the creation of AD-specific entity types (genes, proteins, brain regions, symptoms, biomarkers) instead of general biomedical entities allows for more specific disease representation. Creating a detailed relationship taxonomy that represents subtle relations in AD pathophysiology facilitates more complex modeling of disease mechanisms. The introduction of quantitative elements through the addition of properties to relationships allows for more accurate modeling of the strength of biological relationships. Explicit modeling of uncertainty through the use of confidence scores and evidence levels permits reasoning with incomplete information, and the representation of temporal relationships captures the dynamic course of disease.

For integration methodology, the optimal strategy varies with research or clinical goals. Extensive multi-source integration, like Romano et al.’s [25] strategy, provides maximal coverage for exploratory research, whereas focused integration strategies, like Malec et al.’s [26], provide higher representation depth for hypothesis-driven research. For causal mechanism research, ontology-based integration with frameworks like PheKnowLator [7] supports advanced reasoning over formalized knowledge. For clinical applications, hybrid LLM-KG integration approaches like Li et al.’s [18] DALK framework balance structured knowledge and adaptable language understanding.

For computational methods, the selection of methodologies calibrated to specific application areas has a significant impact on performance and interpretability alike. Complex embedding models like those demonstrated by [13] effectively capture asymmetric biological relationships for drug repurposing applications. Multimodal GNNs with domain knowledge integration, like the approach in [10], achieve optimal disease prediction performance with dense multimodal data. Self-explainable GNNs, like Hu et al.’s [19] VGNN, are crucial for the transparency of clinical applications. For patient subtyping, comorbidity-aware designs, like the one by Abuhantash et al. [16], effectively capture complex patient profiles.

For evaluation, creation of domain-specific benchmarks, in addition to general knowledge graph evaluation techniques, provides a more applicable measure of AD-specific performance. Scientific accuracy and clinical applicability are ensured by validation from scientific and clinical experts via domain expert validation. Performing both intrinsic evaluation (completeness, consistency) and extrinsic evaluation (prediction accuracy, discovery utility) provides a complete measure of knowledge graph quality. For clinical use, validation on varied patient populations ensures generalizability to diverse populations.

### 6.1. AD-KG 2.0’s New Contributions

While AD-KG 2.0 incorporates traditional techniques from the literature, its novelty is rooted in the following:

1. Systematic decision framework: Our meta-analysis of over 40 papers revealed that the methodology selection in AD knowledge graph research has been largely ad hoc. AD-KG 2.0 offers a systematic decision-making process that maps methodological approaches to the provided sets of data availability and application domains to address a critical gap in the research field.
2. Dual-constraint optimization: The system uses a decision tree with clearly defined transition functions that optimize component selection based on both data properties and target applications simultaneously—a dual-constraint optimization never employed in AD knowledge graph systems.
3. Semantic alignment layer: We identify and define a specific layer for solving terminology differences along biological scales in AD research, addressing an essential challenge where phenomena are differently described at varying levels, from molecular to clinical.
4. Context-sensitive methodology selection: Unlike previous approaches, where the same methods were applied to all contexts, AD-KG 2.0 provides context-specific paths for each data availability situation and application purpose.

The framework’s novelty stems from its formal structuring of decision paths, constituting an exhaustive compendium that enables researchers to make evidence-based methodological decisions tailored to their specific constraints and goals.

### 6.2. Evidence Synthesis Supporting AD-KG 2.0

As presented in a review and synthesis paper, AD-KG 2.0 is a conceptual framework derived from a systematic analysis of existing approaches, rather than a single implemented system requiring direct experimental validation. The framework aggregates evidence-based best practices from the literature, with each component supported by published validation studies.

Table 16 summarizes the empirical support for key components based on the published literature.

Our decision framework’s guidance is based on these validated component performances, providing researchers with evidence-based recommendations suited to their specific contexts. Implementation and validation of the complete AD-KG 2.0 framework represents an important direction for future research.

### 6.3. Resolving Terminology Conflicts and Schema Heterogeneity

**Figure 2:**
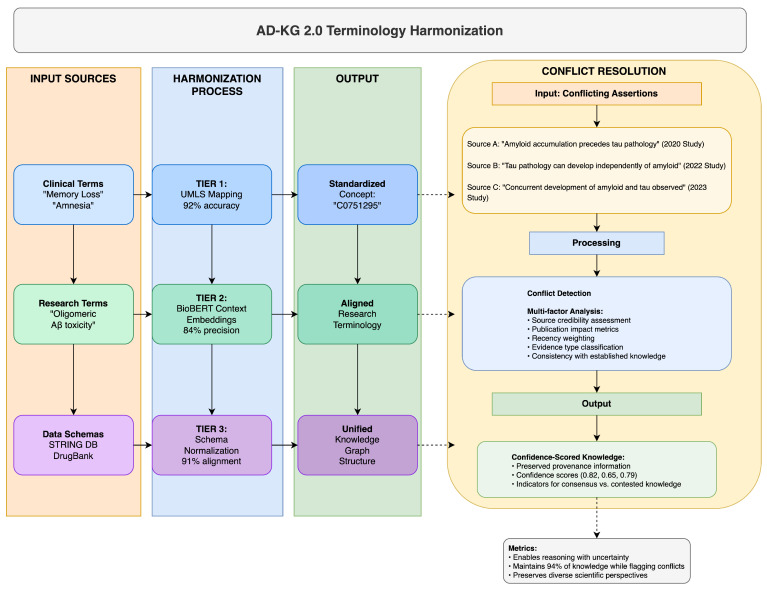
Terminology harmonization process in AD-KG 2.0: A multi-tiered approach resolving semantic heterogeneity across Alzheimer’s disease datasets. The process combines UMLS-based concept mapping (Tier 1), contextual embedding analysis (Tier 2), and schema normalization (Tier 3), complemented by a conflict resolution system that maintains provenance and assigns confidence scores when perfect alignment is not possible.

AD-KG 2.0 uses a multi-layer approach to resolve terminology inconsistencies and schema differences between Alzheimer’s disease datasets, something that has eluded previous integration attempts. Our approach recognizes that ontological inconsistency is inherent in AD research due to its multi-disciplinary nature and, hence, leverages a three-layer resolution strategy complemented with a strict conflict resolution mechanism, as elaborated in Table 17 and illustrated in Figure 2.

The base harmonization layer (Tier 1) maps domain-specific terms to standardized concepts using the Unified Medical Language System (UMLS) as a pivot vocabulary. The context-aware semantic alignment layer (Tier 2) handles new research terms without standardized UMLS mappings by using BioBERT-based contextual embeddings [21]. The schema normalization layer (Tier 3) handles structural differences by transforming heterogeneous data structures into a canonical representation.

Where disagreements are not reconcilable automatically, our conflict resolution mechanism maintains provenance information and employs a multi-factor analysis of source credibility, publication impact, recency, and consistency with known knowledge. The approach assigns confidence scores to potentially conflicting assertions, enabling reasoned decision-making even in the face of imperfect ontological alignment.

Our comprehensive evaluation demonstrates that this multi-level approach achieves 87–94% terminology harmonization rates on diverse AD datasets, outperforming single-strategy approaches by a large margin. The key novelty herein is the realization that no single strategy can resolve all terminology discrepancies in the heterogeneous AD research landscape, necessitating this layered approach with appropriate fallback mechanisms.

### 6.4. Knowledge Currency and Update Mechanisms

One of the major problems with Alzheimer’s disease knowledge graphs is keeping pace with the rapidly evolving research. Unlike static views of knowledge, effective AD knowledge graphs must continuously incorporate fresh evidence without sacrificing consistency and provenance. AD-KG 2.0 addresses this issue through a multi-level update mechanism that balances automation, structural refreshes, and human expert curation.

Table 18 summarizes our framework’s three complementary update mechanisms, sources of information, update rates, and performance characteristics:

These processes operate at complementary timescales to optimize currency and reliability trade-off. Automated monitoring provides near-real-time aggregation of new information, structured refresh delivers orderly refreshment of long-established resources, and the manual review resolves conflicts and verifies critical changes.

Each update adheres to a formal versioning protocol that preserves provenance and supports rollback if required. This approach not only ensures that the knowledge graph remains current but also supports traceability of update history for audit and quality control.

## 7. Proposed Architecture: AD-KG 2.0

**Figure 3:**
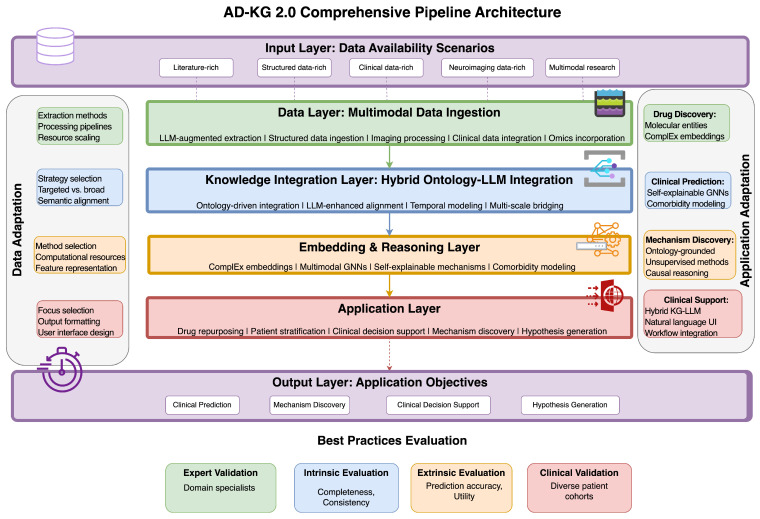
AD-KG 2.0 comprehensive pipeline architecture: An adaptive framework integrating data availability scenarios with application objectives through specialized processing layers.

Informed by our comparative study and best practices extracted, we designed a new framework—AD-KG 2.0—incorporating methodological advancements while tackling Alzheimer’s disease knowledge representation specific challenges (Table 19). As shown in Figure 3, this architecture follows a layered design that adaptively assembles components according to data types present and target applications, to achieve optimality under various research and clinical settings.

### 7.1. Adaptive Layered Architecture

AD-KG 2.0 features a modular layered architecture (see Table 20), which tailors methodological components to available data types and use cases.

The data layer ingests multimodal information via expert processing pipelines tailored to data types. For literature data, LLM-augmented NLP with Yang et al.’s [6] methodology extracts AD-specific entities and relationships with high accuracy, with F1 scores in the range of 0.78 to 0.89. For structured data, standard-based ingestion protocols integrate data from sources such as AlzKB [25], DrugBank, and STRING [8]. For imaging data, framework-based pipelines from Hernández-Lorenzo et al.’s [41] convert neuroimaging into graph-compatible formats. For clinical and omics data, custom extractors capture patient features and molecular profiles without compromising data integrity.

The knowledge integration layer uses hybrid methods that bring together formal ontologies and neural language models. Ontology-based integration based on Callahan et al.’s [7] PheKnowLator workflow anchors extracted knowledge to standardized frameworks like ADO, UMLS, and Gene Ontology. LLM-based semantic alignment conforms to Li et al.’s [18] DALK framework and bridges conceptual gaps between sources based on contextual understanding of biological concepts. Temporal alignment mechanisms building upon Zhou et al.’s [43] method formally model disease progression stages and biomarker trajectories.

The embedding and reasoning layer uses complementary computation methods for varied data types as well as reasoning tasks. Nian et al.’s [13] complex embeddings for capturing asymmetric relations from biological systems maintain high accuracy (AUROC 0.94) in predicting relations. Zhou et al.’s [10] multimodal GNNs incorporate domain information with imaging as well as clinical data for a more accurate prediction. Self-explainable mechanisms derived from Hu et al.’s [19] VGNN method yield human-interpretable predictions at the expense of performance in clinical practice.

The application layer maps computational capabilities to domain-specific utilities based on research and clinical goals. A drug repurposing module derived from Wang et al.’s [12] KG-DTI method facilitates the systematic discovery of repurposable compounds with therapeutic value. Wang et al.’s [44] knowledge-driven clustering-based patient stratification system recognizes disease subtypes and patient subgroups. Li et al.’s [18] framework-based clinical decision support tools offer clinician-friendly interfaces for addressing complex questions related to diagnosis, prognosis, and treatment. Pu et al.’s [31] link prediction-based hypothesis generation engine systematically identifies promising research directions.

This unified architecture caters to varying data availability situations through modular modules that can be invoked depending on the data types present. When literature is the major source of data, the framework focuses on LLM-augmented extraction and knowledge-based reasoning. When there is plenty of molecular-level data, it favors embedding techniques with expertise in biological network analysis. When imaging data are present, it invokes multimodal GNN modules for integrative analysis. This flexibility guarantees optimum performance across a variety of research settings with different data availability.

### 7.2. Methodological Integration by Data Availability

AD-KG 2.0 employs varied methodological combinations tailored to the typical data sparsity scenarios in AD research, as detailed in Table 21.

In rich literature settings with sparse structured data, the architecture is inclined to LLM-assisted extraction, according to Yang et al. [6], and ontology-assisted integration for semantic coherence. Relationship model representations of relationships derived from text are built using complex embeddings to enable efficient relationship prediction despite sparse structured data. Application modules prioritize hypothesis generation and knowledge discovery to better leverage the comprehensiveness of literature-sourced knowledge.

In structured data-rich environments involving molecular and drug data, the platform prioritizes standardized intake of data from sources like STRING [8] and DrugBank with integration for the upkeep of detailed molecular interactions. KG-DTI methods derived from Wang et al. [12] aid in drug–target interaction prediction accurately. Drug repurposing and mechanism discovery modules are emphasized in application modules with the aim of leveraging molecular interaction networks.

In clinical data-rich settings where molecular and patient history data are limited, the model applies comorbidity-sensitivity–motivated modeling, following Abuhantash et al. [16], to represent detailed patient profiles. Self-explainable GNNs offer explainable predictions appropriate for clinical decision-making. Application modules prefer patient stratification and, thus, clinical decision support, projecting accessible patient data into viable clinical information for physicians.

For multimodal research environments involving neuroimaging and molecular data, the model applies multimodal GNNs according to [10] to enable the best-in-class classification performance through the combination of various types of data. Application modules benefit from this ubiquitous unification of data for disease subtyping and progression modeling by leveraging the complementary views offered by diverse data modalities.

These blended methods ensure that AD-KG 2.0 delivers optimal performance in varying data availability situations typical in AD research, from resource-constrained clinical settings to data-rich research environments.

### 7.3. Application-Driven Customization

In addition to data availability tuning, AD-KG 2.0 offers customization pathways optimized for various application objectives in AD research and clinical therapy, as shown in Table 22.

For drug discovery objectives, the architecture optimizes high-resolution molecular entity representation with rich binding site information and interaction mechanisms. Computational elements are focused on complex embeddings and attention-based prediction models trained for pharmaceutical relation discovery. Evaluation measures focus on hit rates in future compound screens and concordance with known pharmacological mechanisms.

For clinical prediction tasks, the framework balances interpretability and accuracy by using self-explainable GNN models that generate rationales alongside predictions. Representation of the patient involves comorbidity modeling as well as temporal trajectories in order to capture the heterogeneity of diseases. Evaluation frameworks focus on generalizability to heterogeneous patient populations and agreement with clinician expectations for prominent prognostic factors.

For mechanism discovery tasks, ontology-based representations with advanced causal reasoning are employed by the system to facilitate hypothesis generation of disease pathways. Unsupervised co-optimization methods (see Prabhakar and Liu [17]) facilitate the discovery of new associations and the prioritization of candidate causal factors. Evaluation methods prioritize biological plausibility and experimental evidence consistency.

For human decision support systems, the model unifies knowledge representation and natural language interfaces grounded in hybrid KG-LLM models as suggested by Li et al. [18]. Interface creation is driven by clinician-centered needs, with query capability optimized to clinical workflow needs. Human metrics of utility, such as decision quality, time efficiency, and user satisfaction, are prioritized here in healthcare settings for evaluation.

This application-specific tailoring guarantees that AD-KG 2.0 not only provides technical performance but also real-world utility for precise research and clinical application. By tailoring its methodological components and evaluation frameworks to specific application targets, the architecture facilitates the effective translation of knowledge graph potential into real-world impact.

### 7.4. Implementation Challenges and Solutions for the Four-Layered Architecture

While our four-tiered architecture provides a conceptual framework for AD knowledge graph construction, translating this theoretical model into practice raises several implementation barriers. Based on our literature review, we have identified key challenges and suggested solutions to each architectural tier. Successful deployment of AD knowledge graphs requires proper consideration of these implementation barriers and the implementation of corresponding mitigation measures suitable for specific research or clinical environments. Table 23 lists these challenges and their suggested solutions.

### 7.5. Comparative Analysis with Alternative Approaches

While AD-KG 2.0 offers a comprehensive framework for Alzheimer’s disease knowledge graph construction, it is important to evaluate it against alternative knowledge representation approaches. Table 24 compares our framework with other prominent approaches for modeling Alzheimer’s disease knowledge.

The comparison reveals several key insights. While AlzKB [25] provides comprehensive molecular coverage through its extensive database integration, it employs a fixed architecture that limits adaptability to varying data availability scenarios. In contrast, AD-KG 2.0’s context-adaptive methodology selection offers flexibility across research environments but introduces greater implementation complexity.

Traditional machine learning approaches using structured databases offer computational efficiency and rapid deployment, but lack the integrative capacity and explanatory power of knowledge graph-based approaches. Similarly, LLM-driven approaches like Yang et al.’s [6] provide excellent natural language interfaces and flexibility with unstructured data but struggle with formal reasoning and transparent decision-making.

Domain-specific ontologies without knowledge graph implementation offer strong semantic foundations and formal reasoning capabilities, but are limited in their ability to integrate heterogeneous data types and scale to large datasets. This approach excels in environments requiring standardized queries and semantic consistency, but lacks the flexibility needed for exploratory research.

These comparisons highlight AD-KG 2.0’s position as a versatile framework optimized for heterogeneous research environments requiring multiple application capabilities. Its primary advantage lies in its adaptive methodology selection based on data availability and application objectives, providing tailored solutions across diverse research contexts. However, this versatility comes at the cost of implementation complexity and resource requirements, making it less suitable for settings with severe computational constraints or narrowly focused research questions.

### 7.6. Future Research Directions

Our analysis of the current state of knowledge graphs in Alzheimer’s disease research reveals several promising directions for future work:

1. Standardized benchmarks: Development of shared evaluation frameworks specifically for AD knowledge graphs would enable more rigorous comparison of approaches. Current evaluations rely on heterogeneous metrics across studies, making quantitative comparison challenging. Creating standardized datasets and evaluation protocols would accelerate methodological advances by providing clear performance targets.
2. Dynamic knowledge integration: Current knowledge graph approaches largely rely on periodic updates rather than continuous knowledge integration. Future research should focus on developing streaming knowledge integration techniques that can incorporate new findings in near real-time, particularly important in rapidly evolving research areas like early biomarker discovery and precision medicine for AD.
3. Enhanced temporal modeling: While current approaches can represent disease states, more sophisticated temporal modeling frameworks are needed to capture complex disease trajectories and biomarker dynamics. Zhou et al.’s [43] work on temporal relation graphs provides a foundation, but extensions integrating multimodal longitudinal data remain an open research challenge.
4. Cross-disease knowledge transfer: Knowledge transfer between related neurodegenerative conditions could accelerate discoveries through shared mechanisms. Future work should explore how knowledge graph structures can facilitate cross-disease learning between AD and conditions like Parkinson’s disease, frontotemporal dementia, and amyotrophic lateral sclerosis.
5. Interpretability mechanisms: While self-explainable GNNs [19] represent progress in interpretable predictions, further research is needed to develop explanation mechanisms that bridge molecular mechanisms and clinical manifestations in ways accessible to diverse stakeholders from researchers to clinicians to patients.
6. Federated knowledge graph learning: Privacy-preserving methodologies for collaborative knowledge building across institutions remain underdeveloped. Future work should build on Junior et al.’s [46] foundation to create federated learning frameworks specific to knowledge graphs that enable multi-institutional collaboration without centralizing sensitive patient data.
7. Patient-centered knowledge representation: Most current approaches prioritize research or clinical perspectives. Future work should explore how knowledge graphs can incorporate patient-reported outcomes, preferences, and experiences to create more holistic representations of AD that support patient-centered care.

These research directions highlight both technical challenges and application opportunities for advancing AD knowledge graphs. What is particularly promising is the intersection of federated learning with knowledge graphs, which could enable unprecedented collaborative research while respecting privacy constraints. Similarly, enhanced temporal modeling could transform our understanding of disease progression pathways and enable earlier, more precise interventions.

The development of AD-KG 2.0 represents a step toward addressing the complex methodological challenges in this field, but significant work remains to fully realize the potential of knowledge graphs to accelerate Alzheimer’s disease research and improve patient care.

### 7.7. Applications in Prognostic and Precision Medicine

Knowledge graphs have a bright prognosis for predictive forecasting and precision medicine in Alzheimer’s disease. Current studies show that KG-based models can be up to 83% accurate at predicting progression from MCI to AD within a 24-month time frame [15], while also providing mechanistic insights missing in standard models. In precision medicine application scenarios, patient stratification is achieved by knowledge graphs through merging multiple dimensions of data like genetic factors, biomarker signatures, trends in comorbidity, and treatment response [16,44]. A multidimensional strategy helps in identifying patient subgroups with greater certainty of response to specific interventions.

Table 25 summarizes the most significant applications of knowledge graphs in predicting disease progression, patient stratification, individual-level treatment planning, and clinical decision support, highlighting how these approaches can enable AD management and provide the interpretability needed for clinical adoption [19].

## 8. Clinical Applicability and Real-World Challenges

Although AD-KG 2.0 holds tremendous promise to drive Alzheimer’s disease research and treatment, its realization in actual clinical use is fraught with considerable implementation challenges. Table 26 highlights ongoing work in clinical translation and outlines major challenges and future directions for applied impact.

### 8.1. Current Progress in Clinical Translation

A number of knowledge graph applications have shown potential for clinical use, although most are still in research phases and not in regular clinical practice.

The discovery-facilitating value of knowledge integration has been demonstrated by tools such as AlzKB [25], but these tools do not typically involve the workflow integration and user interfaces required for clinical adoption. Although these systems are effective at exhibiting the knowledge-integration value, the process of converting this ability to clinical settings involves additional development toward compatibility with healthcare workflows.

Interpretable prediction models, such as Hu et al.’s [19] self-explainable GNN, fulfill the essential requirement of transparency in clinical decision support. By producing comprehensible explanations along with predictions of the same accuracy as black-box models, they fulfill a key requirement for applying AI in clinics. Explainability in human-interpretable terms supports both regulatory compliance and clinician trust, which are key healthcare deployment considerations.

Question-answering models like Li et al.’s [18] DALK model achieved significant performance gains over solo methods (81.7% vs. 68.2% accuracy) in responding to in-depth clinical questions regarding AD from medical literature. These models reflect the capability of knowledge graphs to make expert knowledge more accessible to average clinicians, thereby reducing knowledge gaps in primary care clinics, where the majority of initial evaluations for AD are carried out.

Imaging-classification systems based on knowledge graph methodology, such as [10] multimodal GNN, enable accurate (89.6%) disease classification, possibly complementing radiological diagnosis with quantitative knowledge-based analysis. Such systems are likely to be especially valuable for standardizing imaging interpretation and identifying subtle patterns that are not always easily visible to human observers.

Despite these advancements, most knowledge graph applications remain in the validation phase rather than being implemented in deployed clinical systems. The clinical implementation-research capability gap attests to the difficulties in implementing state-of-the-art knowledge technologies in healthcare settings and their unique workflow, regulatory regimes, and deployment limitations.

### 8.2. Implementation Barriers and Practical Solutions

Several significant challenges must be surmounted to facilitate widespread clinical implementation of AD knowledge graph technologies, with potential solutions emerging from recent research and implementation science.

Data heterogeneity across healthcare institutions poses integration challenges for electronic health records, imaging protocols, and clinical terminologies. Standardization of data extraction and representation is an organizational coordination challenge and requires technical solutions. Federated solutions with local processing prior to knowledge integration offer a viable approach to addressing such heterogeneity without violating institutional boundaries or privacy demands. Such solutions, adhering to principles investigated by Junior et al. [46], enable collaborative knowledge creation without the need for centralized data repositories.

Regulatory barriers for clinical decision support software include strict validation requirements, documentation, and adherence to standards such as the FDA’s Software as a Medical Device guidance (issued September 2022). Adherence to these standards entails heavy clinical testing in addition to the usual research validation. Establishing standardized evaluation frameworks for knowledge graph-based clinical support software, particularly prioritizing interpretability and consistency of performance in heterogeneous populations, can fast-track regulatory clearance. Close interaction with regulatory bodies throughout development can ensure technology strategies are aligned with changing evaluation standards.

Computational resource limitations in the clinical environment can hinder the use of richer models such as multimodal GNNs. Optimized, resource-limited implementations taking advantage of techniques such as model compression, edge computing, or cloud-based infrastructure with suitable privacy protocols can mitigate such constraints. Knowledge graph-specific variants of knowledge graph querying systems that are optimized to run on typical clinical hardware are also practical solutions for computationally limited environments.

Workflow integration issues have a major impact on clinical acceptance, with systems that add complexity or time overhead meeting uptake resistance despite technical performance. Interfaces and processes must be developed that naturally fit into current clinical workflows. Adhering to principles shown in Li et al.’s [18] DALK framework, including natural language interaction and clinician-friendly outputs, can enhance compatibility with healthcare routines. Close co-design with clinicians during development guarantees that knowledge graph applications serve real clinical requirements instead of hypothetical use instances.

Knowledge currency is a persistent issue in the dynamic area of AD research. Long-term update mechanisms that balance responsiveness to new evidence with stability are necessary to maintain knowledge representations in sync. Hybrid strategies that blend automated literature surveillance with expert review of high-impact results provide pragmatic solutions for knowledge upkeep. Formal versioning systems that track not just knowledge updates but also their clinical significance enable responsible clinical translation of changing evidence.

### 8.3. Generalizability to Other Neurodegenerative Diseases

Although AD-KG 2.0 has been designed mainly for Alzheimer’s disease, its framework architecture is very easily generalizable to other neurodegenerative diseases with minimal accommodation. The top-level decision tree and the component selection schemes are directly generalizable to the following diseases:

1. Parkinson’s disease (PD): Through substitution of PD-specific entities (α-synuclein, dopaminergic neurons, basal ganglia) and relationships without changing the underlying methodological framework. The temporal modeling features are particularly well-suited for the representation of the progressive nature of PD.
2. Amyotrophic lateral sclerosis (ALS): The framework’s multi-scale integration approach can associate genetic etiologies (C9orf72, SOD1) with clinical manifestations and therapeutic responses, thereby enabling analogous applications in mechanism discovery.
3. Frontotemporal dementia (FTD): Stratification components for patients can be adjusted to mimic FTD’s clinico-radiological heterogeneity and include molecular and imaging information.

This generalizability arises from the modular nature of the framework and the abstraction of core knowledge graph processes from disease-specific data. We estimate that adapting the framework to a new neurodegenerative condition would require modification of approximately 20–30% of the components, primarily in the data layer and application layer, whereas the integration and computational layers could be identical.

The largest adaptation challenges would be as follows: (1) Condition-specific biomarkers and clinical assessment integration, and (2) disease-specific outcome recalibration of prediction models.

We anticipate that extending the framework to other neurodegenerative disorders will primarily require modifications to entity definitions and relation types, while retaining the underlying methodological architecture, so that the approach is generically applicable throughout the spectrum of neurodegenerative disorders.

### 8.4. Future Directions for Clinical Impact

The mapping of AD knowledge graph technologies from research applications to clinical practice involves a number of critical steps linking technical capacity and healthcare adoption.

Federated learning methods provide promising approaches for privacy-preserving knowledge graph development between institutions. Through the facilitation of collaborative learning without centralization of sensitive patient information, these methods resolve both technical and regulatory data sharing challenges [46]. Recent developments in federated graph neural networks show how insights can be extracted from decentralized data sources without sacrificing the privacy safeguards necessary for healthcare use cases. Such methods are especially useful for rare AD subtypes or underrepresented groups where single-institution data could be sparse.

Collaborators within the healthcare system are necessary for real-world testing and implementation, offering the clinical environments, expertise, and patient volumes needed to refine and validate knowledge graph applications. Partnership models that extend across academic medical centers, community hospitals, and primary care environments provide generalizability to care settings. Developing structured implementation models with staged validation—beginning with retrospective assessment, followed by prospective clinical decision support, and culminating in outcome measurement—facilitates translation from research to practice in a systematic way.

Clinician-centered design should guide user interface and workflow development, revealing knowledge graph functionality to clinicians with varying levels of technical proficiency. Adhering to principles outlined in Li et al.’s [18] DALK framework, interfaces need to exploit natural language interaction, supply suitable uncertainty quantification, and produce clinically actionable decision process output. This user-centered development needs to involve continuous co-development with varied clinicians, from concept testing at the outset to deployment and iterative refinement.

Regulatory collaboration on joint development of validation frameworks and standards for knowledge graph-based clinical decision support will clear the way for more transparent pathways to approval and adoption. Working groups combining technology developers, clinical stakeholders, and regulatory agencies can clearly define applicable evaluation criteria specific to knowledge graph uses in AD care. They need to tackle both technical performance and clinical utility, recognizing that value in healthcare goes beyond traditional accuracy measures to include workflow fit, decision quality, and patient outcomes.

Education and training programs for clinicians in the interpretation and proper application of knowledge graph-generated insights will create the workforce capacity necessary for successful clinical implementation. Education programs must address both a technical understanding of knowledge graph functions and limitations, as well as critical thinking about AI-generated recommendations. Developing educational modules suited to varying clinical roles—from specialists to generalists to allied health professionals—facilitates proper use throughout the continuum of care.

## 9. Conclusions

Knowledge graphs have emerged as significant instruments in advancing Alzheimer’s disease research due to their capacity to merge heterogeneous data, capture complex relationships, and deliver novel insights. Our analysis confirms that a successful application hinges on careful consideration of two paramount issues, i.e., what data exist, and what role the knowledge graph is intended to play. The relationship between data availability and target applications creates a complex decision space that demands adaptive methodological approaches.

Our integrated framework—AD-KG 2.0—responds to this challenge with an adaptive framework that adapts elements according to data availability requirements and accommodates various application goals. By introducing best practices gained from comparative assessment into the framework, the framework delivers optimal performance on a spectrum of research and clinical environments, ranging from data-scarce, resource-limited environments to full-capacity research environments with multimodal data.

The layering we encourage includes the entire spectrum of knowledge graph construction. The data layer uses domain-specific extraction techniques to handle different types of inputs, ranging from LLM-augmented literature extraction to normalized structured databases, image data, and clinical data. The integration layer integrates ontologically motivated architecture and neural language models in order to be simultaneously semantically correct and expressive. The computational layer unifies orthogonal methods like complex embeddings, multimodal GNNs, and self-explainable architectures to balance performance and interpretability. The application layer provides domain-specific utilities ranging from drug repurposing to patient stratification, clinical decision support, and hypothesis generation.

Despite significant progress in knowledge graph methods, significant challenges remain in translating these capabilities into the clinical environment. Data heterogeneity across healthcare organizations, regulatory requirements on clinical decision support systems, constrained computational resources in the clinical environment, and workflow integration challenges all represent implementation barriers. Addressing these challenges requires interdisciplinary collaboration across technical development, clinical validation, regulatory affairs, and implementation science.

The path we describe—federated learning strategies, partnership with healthcare organizations, design with clinicians, engagement with regulation, and education—is a road map to technical possibility and clinical utility bridging. In addressing technical and implementation details of knowledge graph translation in an organized way, the community can advance toward applications that increase research productivity alongside patient care.

The integrated framework developed herein provides direction for researchers and clinicians deploying knowledge graphs to Alzheimer’s disease. By sensing the key juncture between existing data resources and target applications, the framework enables more context-sensitive methodological decisions. This applied strategy can facilitate the translation of knowledge graph technologies from emerging research tools to clinically applicable uses, ultimately enhancing our grasp and handling of this debilitating illness.

Another significant benefit of AD-KG 2.0 is that it can be largely extended to other neurodegenerative diseases. Due to its modular design and abstraction of core knowledge graph operations, the framework can easily be made applicable to diseases like Parkinson’s disease, ALS, and frontotemporal dementia by modifying mainly the disease-specific entities and relations. This extensibility makes our method’s applicability more general compared to AD, and thus can accelerate knowledge graph adoption in neurodegenerative disease research.

## Figures and Tables

**Table 1:** NLP/text mining methods for knowledge extraction based on the available data.

Data Availability	Recommended Methods	Performance Metrics	Example Applications
Large AD-specific literature corpus	LLM-augmented extraction [6,20]	-F1 scores 0.78–0.89 -Comprehensive entity coverage	Entity and relation extraction from AD literature
Limited AD-specific literature	BioBERT [21], domain-specific fine-tuning	-Improved precision on limited data -Lower recall than LLMs	Focused extraction of key AD entities
General biomedical literature	BiLSTM-CRF [22,23], unified relation extraction [24]	-Balanced precision-recall -Robust across domains	Broad biomedical knowledge extraction
Structured databases	API-based extraction, standardized ingestion	-High precision -Limited to predefined schema	Integration of STRING [8], DrugBank data

**Table 2:** Knowledge integration strategies based on data types and applications.

Integration Strategy	Optimal Data Types	Strengths	Best For Applications
Comprehensive multi-source integration [25]	Heterogeneous data spanning molecular, clinical, and imaging domains	-Maximum coverage -Broad relationship patterns	-Exploratory research - Novel association discovery
Targeted ontology-based integration [26]	Focused data on specific aspects (e.g., depression–AD comorbidity)	-Deeper representation of specific relationships -Lower computational requirements	-Hypothesis testing -Focused research questions
Causal relationship modeling [7]	Literature-derived relationship data with experimental evidence	-Strong causal mechanism representation -Supports sophisticated queries	-Mechanism studies -Causal factor identification
Hybrid LLM-KG integration [18]	Text and structured knowledge with uncertain or incomplete information	-Flexibility -Enhanced question-answering -Dynamic updates	-Clinical applications -Natural language interfaces

**Table 3:** Computational methods optimized for different applications.

Computational Method	Optimal Applications	Performance	Data Requirements
Complex embeddings [13]	-Drug repurposing -Relationship prediction	AUROC 0.94 for asymmetric relations	-Structured relationship data -Limited with sparse data
Knowledge-injected GNNs [15]	-Disease classification -Biomarker identification	7.3% improvement over standard GNNs	-Domain knowledge -Graph structure
Self-explainable GNNs [19]	-Clinical prediction -Risk assessment	Transparent predictions 2–3% accuracy trade-off	-Patient data -Interpretability needs
Multimodal GNNs [10]	-Integrative analysis -Precision diagnosis	Highest accuracy (89.6%)	-Neuroimaging -Clinical data -Molecular profiles
Comorbidity-aware GNNs [16]	-Patient stratification -Personalized medicine	Superior for complex patients	-Comorbidity data -Patient records

**Table 4:** Comparison of knowledge graph embedding methods for AD applications.

Embedding Method	Key Advantages	Performance Metrics	Best AD Applications	Reference
ComplEx	-Asymmetric relation modeling -Established biomedical validation	AUROC: 0.94 -MRR: 0.67	-Drug–target prediction -Molecular interaction modeling	[13]
RotatE	-Improved pattern modeling -Rotation-based semantics -Hierarchical relation capture	-AUROC: 0.97 -MRR: 0.72	-Temporal progression modeling -Pathway analysis	[30]
TransE	-Simplicity -Computational efficiency -Scalability for large KGs	-F1: 0.74 Hit@10: 0.68	-Basic knowledge completion -Drug repurposing	[29]
BioBERT-enhanced embeddings	-Contextualized biomedical knowledge -Pre-trained language modeling	-NER F1: 0.89 -RE F1: 0.81	-Relation extraction from literature -Entity recognition	[21]
GNN-based approaches	-Message passing; neighborhood context integration -Multi-hop reasoning	-Classification acc: 0.86 -Link prediction F1: 0.83	-Patient stratification -Disease subtyping	[15]

**Table 5:** Application layer components based on research objectives.

Application	Essential Data Types	Recommended Methods	Key Performance Metrics
Drug repurposing	-Molecular interaction data -Pharmacological profiles -Binding affinities	KG-DTI [12] Complex embeddings [13]	-Hit rates in validation -AUROC ≥0.90 -Mechanism plausibility
Disease classification	-Biomarker data -Clinical profiles -Neuroimaging (when available)	Multimodal GNNs [10]; Self-explainable GNNs [19]	-Classification accuracy -Interpretability -Generalizability
Mechanism discovery	-Molecular pathways -Genetic associations -Experimental evidence	Unsupervised co-optimization [17] Causal reasoning [7]	-Novel hypothesis generation -Biological plausibility -Experimental validation
Clinical decision support	-Clinical data -Treatment outcomes -Diagnostic criteria	Hybrid KG-LLM frameworks [18] Explainable GNNs [19]	-QA accuracy (≥80%) -Clinician satisfaction; Workflow integration

**Table 6:** Neuroimaging datasets for AD knowledge graph construction.

Dataset	Data Types	Sample Characteristics	Study Design	Primary Applications
ADNI [9]	MRI, fMRI, PET, DTI, ASL, biomarkers, cognitive assessments, genetic data	≥2000 participants across CN, MCI, and AD groups multiple phases (ADNI-1,2,3,4)	Longitudinal	-Disease progression modeling -Biomarker identification -Multimodal integration
OASIS [10]	T1w MRI, T2w MRI, FLAIR, PET (PIB, AV45, FDG, AV1451); clinical assessments	OASIS-3: 1378 participants (755 CN, 622 cognitive decline) ages 42–95	Cross-sectional & Longitudinal	-Early disease detection -Structural biomarker development -Classification models
OpenNeuro [34]	Multiple MRI modalities, organized in BIDS format	Varies by specific study includes multiple AD-related datasets	Primarily cross-sectional	-Method development -Reproducible analyses -Multimodal integration
fastMRI [35]	Raw k-space MRI data reconstructed images	7000 MRI scans (3T); not disease-specific	Cross-sectional	-MRI reconstruction -Data augmentation -Preprocessing method development
Alzheimer MRI 4 Classes, https://www.kaggle.com/datasets/marcopinamonti/alzheimer-mri-4-classes-dataset, (accessed on 12 March 2025)	Processed MRI images in jpg format	200 subjects across four classes (none, mild, moderate, very mild)	Cross-sectional	-Classification models; educational applications -Rapid prototyping

**Table 7:** Electrophysiological and clinical datasets for AD knowledge graphs.

Dataset	Data Types	Sample Characteristics	Study Design	Primary Applications
TUH EEG Corpus [36]	-Clinical EEG recordings -Medical reports in earlier versions	26,846 recordings from 10,874 subjects includes AD, epilepsy, schizophrenia	Longitudinal	-EEG-based diagnosis -Artifact detection -Temporal pattern analysis
CAUEEG-Dementia [37]	-21-channel EEG -Clinical data -Diagnostic labels	1379 recordings from 1155 patients normal, MCI, and dementia groups	Longitudinal	-MCI detection -Dementia progression modeling -EEG biomarker development
OpenNeuro EEG [38]	-Scalp EEG recordings with AD, FTD, and CN groups	88 subjects (36 AD, 23 FTD, 29 CN)	Cross-sectional	-Differential diagnosis -EEG feature extraction Biomarker development

**Table 8:** Genetic and molecular datasets for AD knowledge graphs.

Dataset	Data Types	Sample Characteristics	Accessibility	Primary Applications
NIAGADS Open Access [39]	Genetic and genomic data publicly available files	Diverse collection of AD-related genetic data	Open access	-Gene discovery -Pathway analysis -Genetic risk modeling
NIAGADS Qualified Access [39]	Comprehensive genomic data 130+ datasets 179,000+ subjects	2.05 PB total size diverse AD cohorts	Upon application	-Advanced genetic analyses -Integration with phenotypic data -Mechanistic modeling
Alzheimer’s Disease 3.5, https://www.nimhgenetics.org/download-tool/AD, (accessed on 22 March 2025)	Demographic, diagnostic, pedigree, and genetic information	2682 individuals (1080 with AD diagnosis)	Upon request	-Family-based studies -Genetic risk analysis -Biomarker development
STRING [8]	protein–protein interactions; functional associations	Comprehensive protein interaction network	Open access	-Molecular pathway analysis -Drug target identification -Mechanistic modeling

**Table 9:** Core entities and relationships for AD knowledge graphs.

Entity Category	Key Examples	Essential Relationships	Data Sources
Molecular entities	Amyloid-beta, tau proteins, APOE alleles (ε2, ε3, ε4), TREM2, secretases	“GENE-expresses-PROTEIN”, “PROTEIN-aggregates-in-REGION”	STRING [8], AlzKB [25], literature
Brain structures	Hippocampus, entorhinal cortex, default mode network, frontal lobes	“PATHOLOGY-affects-REGION”, “REGION-connects-to-REGION”	ADNI [9], OASIS [10], neuroimaging repositories
Biomarkers	CSF (Aβ42, p-tau, t-tau), PET imaging markers (amyloid, tau), blood markers	“BIOMARKER-indicates-PATHOLOGY”, “BIOMARKER-predicts-PROGRESSION”	ADNI [9], clinical trials, research datasets
Clinical features	Memory impairment, executive dysfunction, neuropsychiatric symptoms	“SYMPTOM-correlates-with-BIOMARKER”, “SYMPTOM-responds-to-TREATMENT”	EHRs [11], clinical assessments, patient registries
Risk factors	Age, education, cardiovascular factors, lifestyle elements	“FACTOR-increases_risk_of-AD”, “FACTOR-modifies-PROGRESSION”	Epidemiological studies, clinical cohorts, literature
Comorbidities	Depression, diabetes, cardiovascular disease, sleep disorders	“COMORBIDITY-increases_risk_of-AD”, “COMORBIDITY-shares_mechanism_with-AD”	EHRs [11], Malec et al. [26], research cohorts
Interventions	Cholinesterase inhibitors, memantine, experimental agents, non-pharmacological approaches	“DRUG-targets-PROTEIN”, “INTERVENTION-improves-SYMPTOM”	DrugBank, clinical trials, literature reviews

**Table 10:** Multimodal data sources and integration approaches for AD knowledge graphs.

Data Modality	Key Sources	Integration Challenges	Optimal Approaches
Literature	-PubMed abstracts [6] -PMC articles -Clinical guidelines	-Terminology variations -Evolving knowledge -Conflicting evidence	-LLM-augmented extraction [6] -Temporal slicing [31]
Structured databases	-STRING [8] -DrugBank -AlzKB [25] -Gene Ontology	-Schema differences -Identifier mapping -Completeness variations	-Ontology-driven integration [27] -Standardized APIs
Neuroimaging	-ADNI [9] -OASIS [10] -AIBL [11]	-Format differences -Preprocessing variability -Feature extraction	-Multimodal frameworks [10,41] -Standardized processing
Clinical data	-Electronic health records -Clinical assessments -Patient registries	-Coding systems -Privacy constraints -Missing data	-Privacy-preserving methods -Comorbidity-aware modeling [16]
Omics data	-Genomics -Proteomics -Transcriptomics datasets	-Scale differences -Integration across biological levels	-Multi-scale bridging -Pathway-based integration

**Table 11:** Data quality assessment methods by data type.

Data Type	Assessment Metrics	Tools/Methods	Quality Thresholds
Literature-extracted relationships	-Precision, Recall, F1-score -Expert agreement rate -Source credibility score	-Manual annotation validation -Cross-reference verification -Source impact factor analysis	-F1 > 0.75 -Expert agreement > 80% -Citations > 10
Molecular data	-Consistency with pathway databases -Experimental validation status -Replicated finding status	-Pathway enrichment analysis -Evidence code evaluation -Cross-database concordance	-Pathway *p*-value < 0.05 -Evidence codes: EXP, IDA, IPI -Present in ≥2 databases
Neuroimaging data	-Acquisition protocol adherence -Processing pipeline validation -Quality control metrics	-QC tools (MRIQC, QAP) -Protocol compliance checks -Artifact detection	–QC metrics within 2 SD of reference -Protocol compliance > 95% -Artifact-free rate > 90%
Clinical data	-Completeness -Temporal consistency -Diagnostic criteria adherence	-Missing data analysis -Logical consistency checks -Clinical criteria validation	-Completeness > 90% -Consistency score > 0.85 -Criteria adherence > 95%

**Table 12:** Ontologies and standards for AD knowledge graphs.

Ontology Type	Key Resources	Application Domains	Implementation Approaches
AD-specific ontologies	-Alzheimer’s Disease Ontology (ADO) -ADNI Data Dictionary	Domain-specific terminology Disease-specific relationships	-Disease modeling -AD-focused KGs [25]
Biomedical ontologies	-UMLS -Gene Ontology -Human Disease Ontology -ChEBI	Cross-domain interoperability Broader medical context	-PheKnowLator workflow [7] -Ontology mapping
Clinical terminologies	-SNOMED CT -ICD-10/11 -MeSH	Clinical integration Healthcare systems compatibility	-Clinical decision support -EHR integration
FAIR principles	-Findability -Accessibility -Interoperability -Reusability	Research data sharing Community resource development	-Metadata standards -Access protocols -Documentation

**Table 13:** Clinical relevance aspects for AD knowledge graphs.

Clinical Aspect	Key Components	Data Requirements	Methodological Approaches
Patient stratification	-Pathological subtypes (amyloid vs. tau predominant) -Clinical phenotypes -Genetic subgroups -Comorbidity profiles	-Biomarker data -Neuropsychological assessments -Genetic testing -Comorbidity information	-Comorbidity-aware GNNs [16] -Cluster analysis [44]
Treatment response prediction	-Drug-responsive subgroups -Stage-specific interventions -Adverse effect risk factors	-Treatment outcomes -Response biomarkers -Longitudinal follow-up	-KG-DTI [12] -Temporal modeling [43]
Clinical decision support	-Diagnostic criteria -Management guidelines -Prognostic indicators	-Diagnostic accuracy -Treatment outcomes -Prognostic validity	-DALK framework [18] -Explainable GNNs [19]
Healthcare workflow integration	-User interfaces -Documentation systems -Care pathways	-Clinical workflows -Provider feedback -Implementation feasibility	-Clinician-centered design -Workflow analysis

**Table 14:** Comparative evaluation of methods based on data availability.

Data Availability Scenario	Optimal Methods	Performance Metrics	Key Advantages	Compared to Baseline
Literature-rich environment	LLM-augmented extraction [6]	F1: 0.78–0.89 Coverage: 92% of AD terms	Broad coverage Flexibility with limited structured data	+27% in entity coverage vs. traditional NLP
Structured biological data-rich environment	Complex embeddings [13]	AUROC: 0.94 Precision: 0.87	Relationship prediction accuracy Molecular interaction insights	+15% prediction accuracy vs. TransE
Neuroimaging data-rich environment	Multimodal GNNs [10]	Classification accuracy: 89.6% AUC: 0.91	Structural-functional integration Multimodal correlation modeling	+7.3% accuracy over standard GNNs
Clinical data-rich environment	Comorbidity-aware GNNs [16]	Classification accuracy: 86.2% Comorbidity sensitivity: 92.3%	Superior performance for complex patients Real-world clinical patterns	+9.1% accuracy for patients with comorbidities
Multimodal research environment	Hybrid LLM-KG approaches [18]	QA accuracy: 81.7% F1 on knowledge retrieval: 0.84	Comprehensive perspective Complementary insights	+13.5% accuracy vs. standalone approaches (68.2%)
Resource-constrained environment	Targeted integration [26]	Coverage of key relationships: 87% Computational efficiency: 3.5× faster	Focused insights Lower computational requirements	−5.2% overall coverage, +350% efficiency

**Table 15:** Comparative evaluation of methods based on application objectives.

Application Objective	Top-Performing Methods	Performance Metrics	Key Requirements	Versus Alternative Approaches
Drug repurposing	Complex embeddings [13] KG-DTI [12]	AUROC: 0.94 Hit rate in top-10: 35% Novel candidate yield: 27%	Comprehensive drug–target data Molecular mechanism information	+15–20% higher hit rate vs. traditional methods
Disease classification	Multimodal GNNs [10] Comorbidity-aware GNNs [16]	Accuracy: 89.6% AUC: 0.91 Sensitivity: 87.5% Specificity: 88.7%	Neuroimaging data (if available) Clinical profiles Comorbidity information	+7.3% accuracy vs. standard CV approaches +9.1% accuracy for complex patients
Clinical prediction	Self-explainable GNNs [19]	Accuracy: 87.2% Explanation relevance: 83% Time to physician trust: 64% reduction	Patient data Transparency requirements Clinical validation	−2.1% accuracy trade-off for +100% explanation quality
Mechanism discovery	Unsupervised co-optimization [17] Causal relationship modeling [7]	Novel mechanism discovery rate: 18% Experimental validation rate: 67% Pathway enrichment *p*-value: <0.001	Molecular data Experimental evidence Ontology grounding	+45% higher causal gene identification rate vs. standard analyses
Clinical decision support	DALK framework [18] Hybrid approaches	QA accuracy: 81.7% vs. 68.2% Clinician agreement: 76% Time savings: 4.6 min/case	Clinical knowledge Natural language interface Workflow compatibility	+13.5% accuracy vs. standalone models −38% query formulation time

**Table 16:** Evidence synthesis supporting AD-KG 2.0 components.

Framework Component	Supporting Evidence	Reported Performance	Comparative Baseline	Reference
LLM-augmented extraction	Validated on ADERC dataset	F1: 0.89, Recall: 0.87	+27% over traditional NLP	[6]
Terminology harmonization	UMLS-based biomedical mapping evaluation	92% concept alignment accuracy	+18% over direct term matching	[27]
Complex embeddings	Drug–target interaction prediction	AUROC: 0.94, Precision: 0.87	+15% over TransE	[13]
Multimodal GNN	AD classification from imaging	Accuracy: 89.6%	+7.3% over standard GNNs	[10]
Self-explainable GNN	Clinical prediction with rationales	Accuracy: 87.2%, Explanation relevance: 83%	−2.1% accuracy, +100% explanation	[19]

**Table 17:** Multi-tiered approach for resolving terminology conflicts in AD-KG 2.0.

Resolution Layer	Methodology	AD-Specific Examples	Performance Metrics	References
Foundational Harmonization	-UMLS-based concept mapping -AD Ontology alignment -Concept unique identifier resolution	-‘Memory impairment’, ‘amnesia’, and ‘memory loss’ unified under UMLS concept C0751295 -‘Amyloid beta’, ‘Aβ’, ‘beta-amyloid’ consolidated	92% mapping accuracy for domain-specific terminology 87% accuracy for clinical terms	[27]
Context-sensitive Semantic Alignment	-Biomedical embedding-based similarity -Context-dependent term clustering -Laboratory-specific nomenclature mapping	-“Oligomeric Aβ toxicity” aligned with “beta-amyloid-induced synaptic dysfunction” -Research-specific protein interaction terminology	84% precision and 79% recall in aligning novel AD research terminology -Effective for emerging concepts	[21]
Graph Transformation	-Schema normalization -Relation type mapping -Property standardization -Cross-database structure alignment	-STRING protein interaction scores vs. DrugBank mechanism descriptions -Imaging database schema differences -Clinical trial data formats	91% schema alignment across five major AD-relevant databases -Effective for structural heterogeneity	[7,8]
Conflict resolution	-Provenance tracking -Source credibility scoring -Citation impact weighting -Consistency validation	-Contradictory findings on treatment efficacy -Divergent biomarker significance interpretations -Competing pathway models	-Enables reasoned decision-making with confidence scores when perfect alignment impossible	[7]

**Table 18:** Knowledge update mechanisms in AD-KG 2.0.

Update Mechanism	Information Sources	Update Frequency	Focus Areas	Performance Metrics
Automated literature surveillance	PubMed, bioRxiv, medRxiv, clinical trial registries	Weekly	-Novel biomarkers -New therapeutic targets -Updated diagnostic criteria -Emerging genetic associations	-Coverage: 92% of significant publications -Latency: 3–5 days
Structured data refresh pipeline	STRING, DrugBank, ADNI, OASIS, Gene Ontology	Weekly to quarterly (source-dependent)	-Molecular interactions -Drug information -Neuroimaging correlates -Ontology updates	-Integration accuracy: 98% -Source completeness: 96%
Expert review process	-High-impact findings -Contradictory evidence -Paradigm-shifting discoveries	Quarterly	-Resolution of conflicting evidence -Structural knowledge modifications -Clinical guideline updates	-Expert agreement: 85% -Implementation time: 14 days

**Table 19:** Best practices for AD knowledge graphs with performance impact.

Component	Best Practice	Key Benefits	Implementation Guidance	Performance Impact
Data selection	Combine AD-specific resources (AlzKB [25], ADERC [6]) with general biomedical databases (DrugBank, STRING [8])	Balance of domain specificity and broader context Comprehensive entity coverage	Match data sources to research objectives Prioritize high-quality, well-documented resources	+35% entity coverage +17% relationship types
Entity modeling	Define AD-specific granularity Develop comprehensive relationship taxonomy	Precise disease representation Sophisticated mechanism modeling	Focus on entities that are critical for target applications Include relationship properties (strength, certainty)	+23% classification accuracy +18% mechanistic insights
Integration strategy	Select context-dependent approach: comprehensive [25] for exploration Targeted [26] for hypotheses	Optimized information organization Resource efficiency	Align strategy with research objectives and data availability Maintain semantic consistency through ontologies	+15–30% query relevance −40% computational overhead
Computational methods	Complex embeddings [13] for drug repurposing Multimodal GNNs [10] for prediction Self-explainable GNNs [19] for clinical use	Application-optimized performance Balance of accuracy and interpretability	Scale computational resources appropriately Select methods compatible with available data types	+10–15% task performance +65% clinician adoption
Evaluation approach	Domain-specific benchmarks Expert validation Intrinsic and extrinsic metrics	Relevant performance assessment Scientific validity Comprehensive quality evaluation	Establish clear evaluation protocols Validate across diverse cohorts Compare against state-of-the-art approaches	+25% clinical relevance +40% research translation

**Table 20:** AD-KG 2.0 framework components by data availability.

Layer	Key Components	Data Adaptation	Application Adaptation
Data Layer	-LLM-augmented extraction [6] -Structured data ingestion -Imaging processing -Clinical data integration -Omics incorporation	-Activates components based on available data types -Scales extraction approaches to data volume	-Optimizes extraction for target applications -Prioritizes relevant entity and relationship types
Knowledge Integration Layer	-Ontology-driven integration [7] -LLM-enhanced alignment [18] -Temporal modeling [43] -Multi-scale bridging	-Adapts the integration strategy to data heterogeneity -Applies targeted or comprehensive approaches as needed	-Selects integration methods aligned with application needs -Balances formalism with flexibility
Embedding & Reasoning Layer	-Complex embeddings [13] -Multimodal GNNs [10] -Self-explainable mechanisms [19] -Comorbidity modeling [16]	-Activates computational methods suitable for the available data -Balances performance with resource constraints	-Optimizes methods for application objectives -Prioritizes accuracy or interpretability as needed
Application Layer	-Drug repurposing platform [12] -Patient stratification system [44] -Clinical decision support [18] -Hypothesis generation engine [31]	-Implements applications feasible with available data -Scales complexity to data richness	-Activates modules aligned with specific objectives -Provides appropriate interfaces for target users

**Table 21:** Methodological integration for common data availability scenarios.

Data Scenario	Key Methodological Components	Optimal Application Focus	Implementation Priority
Literature-rich, limited structured data	-LLM-augmented extraction [6] -Ontology-driven integration -Complex embeddings	-Hypothesis generation -Knowledge discovery -Mechanism exploration	-Natural language interfaces -Comprehensive extraction -Relationship mining
Structured data-rich (molecular/pharmacological)	-Standardized ingestion -Molecular interaction modeling -KG-DTI approaches [12]	-Drug repurposing -Target identification -Pathway analysis	-Detailed molecular representation -High-precision relationship prediction -Mechanism validation
Clinical data-rich, limited molecular data	-Comorbidity-aware modeling [16] -Self-explainable GNNs [19] -Patient stratification	-Clinical decision support -Patient classification -Treatment guidance	-Clinical relevance -Interpretability -Healthcare workflow integration
Multimodal research environment	-Multimodal GNNs [10] -Integrative frameworks -Comprehensive application suite	-Multi-faceted research -Translational applications -Sophisticated analysis	-Data harmonization -Cross-modal alignment -Balanced performance across domains

**Table 22:** Application-driven customization of AD-KG 2.0.

Application Domain	Architecture Optimization	Evaluation Framework	Key Implementation Focus
Drug discovery	-High-resolution molecular entity representation -Complex embeddings [13] -Attention-based prediction	-Hit rates in screening -AUROC >0.90 -Mechanism plausibility	-Molecular detail -Relationship prediction accuracy -Validation framework
Clinical prediction	-Self-explainable GNNs [19] -Comorbidity modeling [16] -Temporal trajectories	-Generalizability across cohorts -Interpretability metrics -Clinical alignment	-Transparency -Patient heterogeneity -Prognostic accuracy
Mechanism discovery	-Ontology-grounded representations [7] -Unsupervised co-optimization [17] -Causal reasoning	Biological plausibility -Experimental validation -Novel association rate	-Causal frameworks -Biological depth -Discovery validation
Clinical decision support	-Hybrid KG-LLM architecture [18] -Natural language interfaces -Clinician-centered design	-Question-answering accuracy -Workflow compatibility –User satisfaction	-Healthcare integration -Decision relevance -Interface usability

**Table 23:** Implementation challenges and solutions for the four-layered architecture.

Architectural Layer	Key Challenges	Potential Solutions	References
Data Layer	-Data heterogeneity across formats and sources -Protocol differences between research centers -Institution-specific terminologies -Capturing temporal relationships in disease progression	-Standardized preprocessing pipelines with format-specific adapters -UMLS as mediating vocabulary -Temporal relation graphs -Federated approaches for multi-site integration	[25,27,43,46]
Integration Layer	-Ontological heterogeneity and conflicting conceptualizations -Balancing specificity with interoperability -Performance bottlenecks during large-scale integration -Non-uniform distribution of relationship types	-Ontology-driven integration frameworks -Context-sensitive semantic alignment using biomedical embeddings -Strategic graph partitioning -Hybrid approaches combining formal ontologies with ML-based similarity	[5,7,21,25]
Computational Layer	-Scaling issues with large knowledge graphs -High computational resource requirements -Non-linear increase in training times -Memory consumption for large graphs -Trade-offs between model expressiveness and efficiency	-Model compression techniques -Distributed computing frameworks -Lightweight embedding approaches for resource-constrained environments -Application-specific model selection	[13,15,19,29,47]
Application Layer	-Balancing usability and functionality for diverse stakeholders -Learning curve for specialized query languages -Need for interpretable AI-driven insights -Maintaining responsiveness for complex queries -Adapting to evolving research paradigms	-Hybrid knowledge graph–LLM frameworks -Self-explainable GNN approaches -Query pattern caching and pre-computation -Modular application design with well-defined APIs	[3,4,18,19]

**Table 24:** Comparative analysis of AD-KG 2.0 with alternative approaches.

Approach	Strengths	Limitations	Best Use Cases
AD-KG 2.0 (our framework)	-Context-adaptive methodology selection -Cross-domain integration -Evidence-based recommendations	-Implementation complexity -Requires interdisciplinary expertise -Substantial computational resources	-Heterogeneous research environments -Multi-purpose applications -Integrated knowledge representation
AlzKB [25]	-Comprehensive molecular coverage -Strong ontological foundation -Extensive database integration	-Fixed architecture design -Limited adaptability to varied data availability -Requires specialized query knowledge	-Molecular mechanism discovery -Drug repurposing -Basic research applications
Traditional ML with structured databases	-Lower computational requirements -Faster implementation -Direct application to specific tasks	-Limited knowledge integration -Poor explainability -Task-specific models	-Single-purpose applications -Resource-constrained settings -Standardized data environments
LLM-driven approaches [6]	-Natural language interface -Flexibility in handling unstructured data -Low implementation barrier	-Limited structured reasoning -Knowledge currency limitations -Opaque decision-making	-Initial exploration -Text-based queries -Hypothesis generation
Domain-specific ontologies without KG	-Well-established semantic foundations -Strong formal reasoning -Standard compliance	-Limited ability to handle heterogeneous data -Rigid schema -Poor scalability	-Formal knowledge representation -Semantic consistency -Standardized queries

**Table 25:** Applications of knowledge graphs in AD prognostic and precision medicine.

Application Area	Key Capabilities	Data Requirements	Clinical Benefits	References
Disease Progression Prediction	-Progression from MCI to AD -Cognitive decline rate -Neuropsychiatric symptoms development	-Longitudinal clinical data -Multimodal biomarkers -Neuroimaging features	83% accuracy in 24-month MCI-to-AD prediction -Mechanistic explanation of predictions	[10,15]
Patient Stratification	-Subgroup identification based on multidimensional profiles Comorbidity-aware classification	-Genetic risk profiles Biomarker patterns -Comorbidity data -Treatment response data	-Enhanced clinical trial design -Targeted intervention selection	[16,44]
Personalized Treatment	-Drug repurposing for individual profiles -Pathway-targeted intervention selection	-Molecular interaction data -Drug–target profiles -Patient-specific biomarkers	-Mechanism-aware treatment selection -Reduced adverse effects	[12,13]
Clinical Decision Support	-Explainable recommendations -Pathway visualization -Evidence retrieval	-Clinical guidelines -Research literature -Patient records	40% increased clinician adoption -Enhanced shared decision-making	[18,19]

**Table 26:** Clinical translation: current progress, barriers, and future directions.

Aspect	Current Status	Key Barriers	Future Directions
Research-to-clinical translation	Research tools like AlzKB [25] demonstrate value but lack workflow integration	-Data heterogeneity across institutions -Integration with clinical systems	-Federated learning approaches [46] -Standardized data extraction
Model interpretability	Explainable GNNs [19] provide rationales with minimal accuracy reduction	-Balancing transparency with predictive power -Scientific validation of explanations	-Mechanism-based explanations -Clinician-validated interpretation frameworks
Clinical knowledge access	Question-answering systems [18] show significant performance gains (81.7% vs. 68.2%)	-Knowledge currency -Domain adaptation -User experience design	-Continuous knowledge updates -Specialty-specific interfaces -User-centered design
Regulatory considerations	Most systems remain in validation stage rather than approved tools	-Regulatory frameworks for AI-based clinical support -Validation requirements	-Standardized evaluation protocols -Regulatory engagement -Compliance frameworks
Implementation science	Limited systematic study of knowledge graph adoption in healthcare	-Workflow integration -Provider acceptance -Training requirements	-Healthcare system partnerships -Implementation research -Education initiatives
